# It’s Complicated: Maillard, Umami and Flavor Complexity Are Not Key Factors in Liking of Gray Pea Burgers in a Real Consumption Context

**DOI:** 10.3390/foods15061015

**Published:** 2026-03-13

**Authors:** Iuri Baptista, Agnes Harcevic, Magnus Westling, Åsa Öström

**Affiliations:** School of Hospitality, Culinary Arts and Meal Science, Örebro University, 71260 Grythyttan, Sweden

**Keywords:** protein transition, vegetarian, food service, culinary arts, recipe development

## Abstract

Literature suggests that umami, Maillard reaction, and flavor complexity could contribute to sensorial acceptability of plant-based alternatives, but that was yet to be tested. Two field studies with 612 paying customers evaluating a complete meal were conducted in an operating restaurant in Sweden. In the first study, a gray pea burger (Control) was compared to burgers with added monosodium glutamate (MSG) (Umami), grilled (Maillard), or both grilled and added MSG (Complex). In the second study, a simplified gray pea burger (Control 2) was compared to a grilled burger with MSG and aromatics (Complex 2). Check-all-that-apply (CATA) tests show that participants perceived sensory differences between the samples, but their effects in hedonic ratings were inconclusive; only the Maillard sample was significantly more liked than Control and Complex burgers in Study 1. Although limited to their variables and context, these two experiments indicate that umami, Maillard reaction, and complexity, per se, are not key factors to improve liking and willingness to buy (WTB) of plant-based dishes. These results suggest that rather than trying to emulate sensory characteristics considered associated with meat, future research could prioritize addressing cultural barriers to vegetarian food.

## 1. Introduction

Food production and consumption are major contributors to human environmental impact, including greenhouse gas emissions (GHGE), land and water use, eutrophication, and loss of biodiversity [1]. The substitution of animal for vegetable food products is broadly considered one of the most efficient strategies towards more environmentally sustainable diets [2]. In addition, a high consumption of meat is correlated with some of the noncommunicable diseases (NCDs) that are responsible for 75% of the deaths worldwide [3], including heart attacks, hypertension, and cancer [2]. Among the vegetable options to substitute animal products, pulses, the dry seeds of plants in the legume family, including chickpeas, lentils, peas, and beans, are highlighted as sustainable, accessible, healthy, biodiverse, and protein-rich alternatives [4].

The Food and Agriculture Organization (FAO) for the United Nations (UN) declared 2016 the “International Year of Pulses” (IYP), but pulses’ consumption levels are still historically dropping and are particularly low among high-income populations in North America and Europe [5]. In Europe, per capita consumption of legumes is around 1.5 kilos per year, which provides around 2% of the daily protein intake and only 1% of the energy intake [4]. It is estimated that one kilo of cooked peas grown in Sweden emits around 0.1 kg in CO2 equivalent, while one kilo of beef grown in Sweden emits 300 times more, around 30 kg [6]. Legumes also benefit the soil by fixing nitrogen and decreasing the need for oil-based fertilizers when used for crop rotation, but account for less than 3% of Swedish harvested area [7]. The lack of interest from seed and pesticide companies contributed to maintaining the biodiversity of pulses and their production concentrated in small farms, which offer resilience and adaptation against the climate crisis and disruptive events [4].

Pulses have a low content of fat while being rich sources of fibers, protein, and micronutrients, making them promising allies against malnutrition that ranges from undernutrition and micronutrient deficiency to obesity and NCDs [8]. Despite all the benefits of pulses for the environment, economy, agriculture, and health, the exchange of animal products for vegetable alternatives faces obstacles regarding consumer preference and acceptance [9]. Consumers are attached to meat because they consider it necessary, natural, normal, and nice [10]. So far, most scientific literature has focused on improving production of pulses, without offering solutions to tackle the consumer barriers [11].

Consumption of pulses remains challenged by a lack of knowledge on how to prepare and incorporate them in eating patterns, longer cooking time, flatulence, and anti-nutrients like lectins and phytates [8]. Swedish consumers consider low-processed legumes (cooked lentils, peas, beans) as more sustainable and healthier than meat or ultraprocessed plant-based alternatives that mimic animal products, but not as tasty, fun, easy to prepare or popular as meat [9]. Many reported an intention to increase consumption of legumes but struggle to include them in their routine [9], highlighting the potential role of the food service industry (i.e., restaurants, cafeterias, catering, etc.) in this transition [12]. Besides being a major gatekeeper of what people eat with the increase in meals made out-of-home [13], food service can act as a driver of change by offering examples of tasty pulse-based dishes [14].

Previous research with a diversity of methods and participants indicated that seasoning, umami, meat, onion, and broth flavors are correlated to liking of plant-based products, while bitter, beany, burnt, and cardboard flavors are inversely correlated to it [14,15,16]. Cordelle et al. [17] showed that meat aroma improved liking of wheat and chickpea chunks served with a white sauce and quinoa. Suggestions for how to improve the sensory characteristics of plant-based alternatives include increasing the umami taste [18] and Maillard reaction compounds [19]. Although no study on the effect of Maillard and umami in consumer liking of meals with meat substitutes was found, Jouquand et al. [20] indicated that overall liking of baguettes increased with time of baking and Maillard reaction compounds and Miyaki et al. [21] demonstrated that umami compounds had positive effects on the perception of chicken soup.

It is interesting to note that grilling mushrooms increases their perceived meatiness [22], even though it drastically reduces the umami amino acid content because cooking destroys the amino acids and Maillard reaction consumes them together with simple sugars [23]. This could mean that Maillard reaction is more important to meatiness perception than umami taste. Beyond individual flavor components, studies found positive correlations between sensory complexity—although sensory complexity is not consensually defined—and food liking, particularly in relation to sensory-specific satiety (SSS) over multiple exposures [24,25,26].

While the literature shows that umami, Maillard reaction, and complexity can improve liking of food in general, there is a lack of studies that tested them as strategies to tackle the challenge of improving the flavor of vegetarian meals in a food service context. To address this gap, the present study compared a control gray pea burger with three variants designed to emphasize umami, Maillard reaction and overall flavor complexity. The primary objective was to evaluate whether these sensory modifications could significantly influence consumer perception and liking in a complex meal in a real consumption context. The hypotheses were:

**H1.**  *Participants will perceive sensory differences between the burgers.*

**H2.**  *Liking and willingness to buy (WTB) will increase correlated to the increase in flavor complexity of the samples.*

## 2. Materials and Methods

### 2.1. Procedure and Participants

The research was conducted in May 2025 at the Kårhus restaurang, a venue operated by the student union at Örebro University. By the time of the research, they served à la carte lunch from 11:00 to 13:00 from Tuesday to Thursday with three permanent options of main dishes: meatballs with roasted potatoes, lingonberry jam, pickles, and sauce; schnitzel with roasted potatoes, lemon sauce, and salad; and baked potato with chicken curry or feta cheese (vegetarian) and salad. The average number of guests was around 70 per day and the meal costed 55 SEK (approximately 5 euros) for students and 99 SEK (approximately 9 euros) for non-students. For the research, the regular menu was substituted by a “burger week” event, and the restaurant only offered the experimental menu of a gray pea burger with potato wedges for the discounted price of 29 SEK (approximately 2.6 euros) subsidized by the research project to reach the experimental goal of a hundred participants in each condition.

Participants of both studies were recruited through the Kårhus’ and Örebro University’s social media. The research was advertised as a collaboration between the Kårhus restaurang and the School of Hospitality, Culinary Arts and Meal Science as part of a “recipe study” without disclosing the specific goals of the study. Participants were first informed about the research, menu, and allergenics; then, they paid for their meals and were instructed to scan a QR code that led to a questionnaire hosted at EyeQuestion (Logic8 B.V., Elst, The Netherlands). According to Swedish Law 2003:460, studies with human subjects that do not collect biological material or personal information are exempt from ethical review. Incomplete participations (75 in Study 1 and 48 in Study 2) were not included since they only answered the demographic questions to get the codes for the burgers and abandoned the studies before evaluating the meal.

The questionnaire started with information about the study, the participants’ rights and risks, and asked for their free informed consent. If they consented to participate, they were asked for their gender (female, male, or non-binary), age group (16–25 years old, 26–35 years old, 36–45 years old, 46–55 years old, or 56 years old or more), and if they were a student (yes or no). The restaurant added two questions to the survey: how frequently they ate lunch at the restaurant (every day, every week, every month, every semester, every year or less) and what would make them eat more often at the restaurant (if it was cheaper, healthier, environmentally friendlier, faster, tastier, the menu had more options, their friends also ate there, others).

In Study 1, 470 meals were sold and 395 (84%) complete answers to the questionnaire were collected (168 on the first day and 227 on the second day). In Study 2, 265 meals were sold and 217 (82%) complete answers to the questionnaire were collected. The demographic profile of the respondents in both studies is shown in Table 1.

Once they answered these first questions, they got a 3-digit blinding code assigning them randomly to one of the four burger conditions and picked their meal at the restaurant counter. The between-groups design was chosen for realism; people most commonly eat only one entire burger in a meal, not four pieces of four different burgers. Participants were instructed to eat their lunch wherever and however they desired; they were able to serve themselves tap water for free and buy soft drinks at the restaurant. After they had eaten, they answered to “what did you think of the potatoes wedges?” and “what do you think of the burger?” using a hedonic 7-point scale ranging from “1—Very bad” to “7—Very good”. They also answered to “would you buy this meal if it was on the lunch menu at the Kårhus?” using a 5-point scale ranging from “1—Very unlikely” to “5—Very likely”.

Participants were then asked “How would you describe this burger?” in a check-all-that-apply (CATA) task with the terms: “simple”, “complex”, “meaty”, “filling”, “boring”, “fun”, “dry”, “juicy”, “soft”, “crispy”, “umami”, “salty”, “roasted”, “vegetarian”, “healthy”, “luxurious”, “cheap”, “spiced”, “smoky”, “nutty”, “long aftertaste”, and “sustainable”. A CATA task was preferred over other methods for allowing a larger number of attributes without demanding too much time and attention from participants since they were in their weekday lunch break with all the distractions of a real consumption situation [27]. After submitting their answers, they received a code and were invited to get an ice cream at the restaurant counter.

### 2.2. Materials

Gray pea (*Pisum sativum* L.) was chosen for being a historically forgotten legume crop in Sweden that is currently making a comeback by the hands of small organic farmers [28]. It was also important that a small local industry started producing cooked, grinded, and frozen organic gray pea mince (DelMat, Hällefors, Sweden) and made it easily available for all sizes of food service through one of the major restaurant suppliers in the country (Martin & Servera, Stockholm, Sweden). In addition, previous research characterized the sensory profile of gray peas as pleasantly chewy with nut, sweet, beany, and earthy flavors, highlighting their appropriateness to substitute meat mince in patties and fillings [14,29,30].

**Figure 1:**
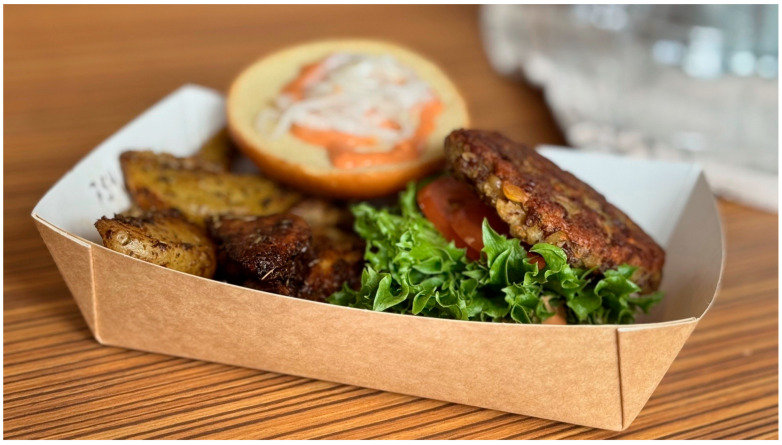
The Maillard burger: Grilled gray pea patty, brioche bun, lettuce, tomato, pickled white onion, and barbecue sauce, served with a side of spiced potato wedges. Photo: Jasenka Dobric.

The gray pea patties in both studies were served in a burger with a toasted bread bun (Korvbrödsbagarn, Örebro, Sweden), lettuce, tomato, white onions, and a vegan barbecue sauce made with mayonnaise (Menigo, Stockholm, Sweden), ketchup (Menigo, Stockholm, Sweden) and liquid smoke (TryMe, New Orleans, LA, USA), as shown in Figure 1. The burger was served with a side of potato wedges seasoned with vegetable oil, salt, dried oregano, dried rosemary, dried thyme, dried onion powder, dried paprika powder, cayenne pepper, and black pepper (all spices from Santa Maria, Mölndal, Sweden). Participants that completed the questionnaire could choose between a chocolate, strawberry, or vanilla ice cream cone (ICA Basic, Solna, Sweden).

#### 2.2.1. Patties—Study 1

The recipes were based on previous research led by one of the authors [14,29,30] and refined for the conditions of this study by two of the authors with chef education and years of experience in professional kitchens. They informally tested dozens of formulations and piloted prototypes with colleagues at the School of Hospitality, Culinary Arts and Meal Science to calibrate the level of each ingredient. The Control patty was composed of gray pea mince, carrot, onion, oat, potato flour, garlic, and salt in the proportions shown in Table 2. In the Umami and Complex patties, half of the salt was substituted by the same weight in monosodium glutamate (Ajinomoto, Balen, Belgium). The Control and Umami patties were baked at 100 °C for 10 min and the Maillard and Complex patties were grilled at 200 °C for 4 min each side.

#### 2.2.2. Patties—Study 2

To address the inconclusive results from Study 1, the patties in Study 2 were modified to become even more “simple” and “complex”. Since addressing the lack of definition of flavor/culinary complexity was not part of the goals of this study, the number of flavor-enriching ingredients and processes were considered a practical proxy for simplicity/complexity of a patty within the context of this study. Therefore, the carrot, onion and garlic were taken out of the Control patty to become Control 2 and the Complex patty gained a mix of spices (paprika, onion powder, cummin, cayenne, black pepper, dried parsley) (Santa Maria, Mölndal, Sweden) and liquid smoke (TryMe, New Orleans, LA, USA) to become Complex 2. The proportions of ingredients in both patties are shown in Table 2.

### 2.3. Statistical Analysis

For each study, a multiple linear regression was performed to regress the three dependent variables (potato liking, burger liking, willingness to buy the meal) on the independent variables (Control, Maillard, Umami, Complex, Control 2, and Complex 2) while controlling for potentially confounding variables (gender and age). The regressions were performed in RStudio version 2025.05.1 + 513 (Posit PBC, Boston, MA, USA). The CATA results were analyzed by comparison of proportions. To improve visualization of the CATA results, a correspondence analysis (CA) was performed in Study 1 but not in Study 2 because it had only two samples. The comparison of proportions and the CA were performed on EyeOpeneR (Logic8 B.V., Elst, The Netherlands). All analyses were performed at a 5% level of significance.

## 3. Results

### 3.1. Study 1

**Figure 2:**
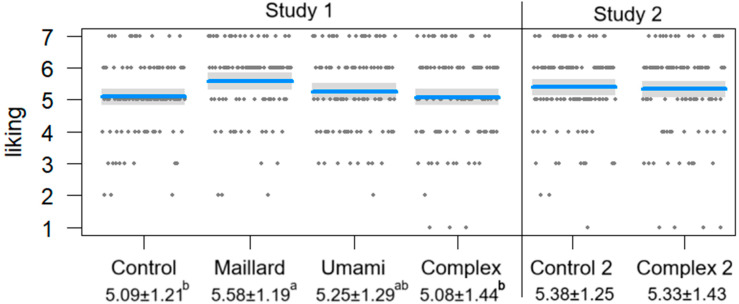
Means (blue lines), standard deviations (gray areas), and distributions (gray dots) of liking ratings for the six burgers in Studies 1 and 2. Values show mean liking and standard deviations. When marked with the same subscribed letter, the values are not significantly different at a 5% level of significance.

**Figure 3:**
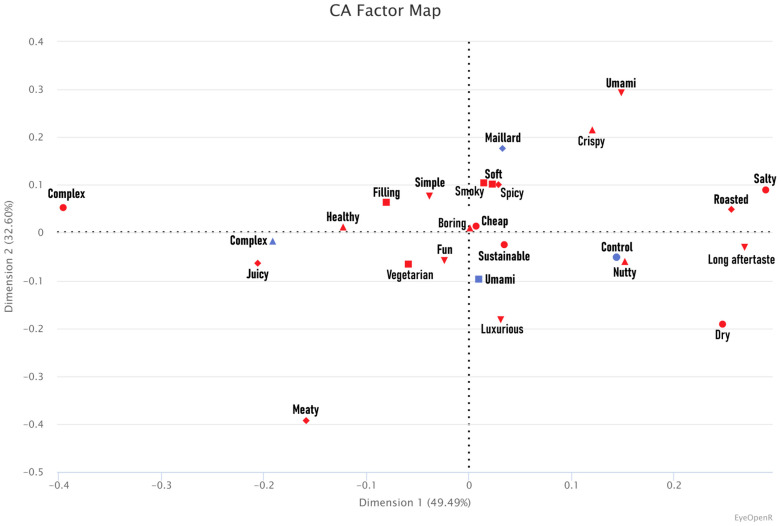
Correspondence analysis (CA) factor map showing the check-all-that-apply (CATA) terms (red markers) associated with the samples Control, Maillard, Umami, and Complex (blue markers) in Study 1.

The multiple linear regression (F [9, 385] = 4.64, multiple R-squared = 0.10, adjusted R-squared = 0.08, *p* < 0.001) showed that the Maillard burger was more liked than the Control (t = 3.05, *p* < 0.05) and Complex burgers (t = 2.60, *p* < 0.05), as shown in Figure 2. It also showed that women liked the burgers more than men (t = 3.17, *p* > 0.001) and participants in age groups 36 to 45 and 46 to 55 years old liked the burgers more than participants in age group 16 to 25 years old (t = 2.92 and t = 3.29, *p* < 0.001 and *p* < 0.001, respectively). No other significant association was found. Table 3 shows the average potato liking, burger liking, and WTB with standard deviation. The comparison of proportions found significant differences between the samples on the terms “dry”, “vegetarian”, and “long aftertaste” as also shown in Table 3. The CA’s first and second dimensions accounted for 49.49% and 32.60% of the data variability, respectively, as shown in Figure 3.

### 3.2. Study 2

The multiple linear regression (F [8, 208] = 0.99, multiple R-squared = 0.04, adjusted R-squared < 0.001, *p* = 0.44) found no significant correlations. The mean liking and rating distribution of the burgers are shown in Figure 2. The comparison of proportions found that Complex 2 was significantly more often described as “crispy”, “spiced”, and “long aftertaste” than the Control 2, as shown in Table 4, which also shows the average potato liking, burger liking, and WTB with standard deviation.

## 4. Discussion

**H1** was confirmed: significant sensory differences were found between the samples in both studies. However, they were few and not directly related to the manipulations and expected outcomes, such as the Complex burgers being more often described as “complex” or the Umami more “umami”, although it is understandable that young students in Sweden would not be able or confident to define and identify them. Overall, taste and aroma terms were less checked by participants compared to “vegetarian”, “healthy”, “sustainable”, “cheap”, and “fun”, or even to texture terms like “dry”, “juicy”, and “soft”. This result indicates that the subsided price, the playfulness of participating in a scientific study, the vegetarian nature of the burgers, and their texture were more important than taste and aroma for the participants.

**H2** was not confirmed: the different degrees of sensory complexity did not have a comprehensive effect on liking or WTB of the burgers. While grilling significantly increased the liking of the Maillard burger compared to Control in Study 1, it did not have the same effect in the Complex burgers from both Studies 1 and 2. It could be some unexpected interaction between grilling and MSG, but this research has no evidence of that. Looking at the CATA results, the dryness of Control and “vegetarianess” of Complex could explain why they were less liked than the Maillard burger, but it is hard to explain why the dryness of Umami was not as penalized or why Maillard was less often perceived as vegetarian than Complex. The difference, then, is more likely to be a type I error or due to individual differences among groups that, unlike gender and age, were not measured and not included as confounders in the regression (e.g., dietary profiles, meat attachment, hunger level, attitudes towards plant-based meals). The low adjusted R^2^ indicates that the model poorly represents the data variability and contributes to this interpretation.

A clearer rejection of **H2** comes from Study 2. Complex 2 was more often described as “filling”, “fun”, “soft”, “crispy”, “spiced”, and “long aftertaste” and less “simple”, “dry”, “nutty”, and “sustainable” than Control 2 (although many of the differences did not reach significance) and both had almost identical mean liking and similar WTB. In this research, the flavor of the patties was not a key factor in consumer liking of vegetarian burgers. In fact, the same is probably true for meat patties as well, since the many popular fast food burger chains have standardized bland patties and rely on other elements, like sauce, to deliver flavor. The results do not mean that a bad tasting patty would not strongly affect burger liking, they only suggest that a lack of umami or Maillard, per se, is not holding back the popularity of vegetarian meals [18,19].

This interpretation resonates with a previous study that could not find a correlation between the likability of vegetarian dishes and their complexity or sensory profile: a light and fresh dish (pasta with pesto and toasted beans) was as liked as a fulfilling and hearty dish (gray pea-filled potato dumpling with brown butter) [14]. Since the patties were just one element in a sandwich with bread, lettuce, tomatoes, pickled onions, and barbecue sauce, just like in a real consumption context, the results also do not contradict previous research that showed that umami, Maillard and complexity can improve palatability of isolated products [19,20,22,25]. Again, the results only show that umami, Maillard, and complexity are not going to boost the popularity of vegetarian meals by themselves.

Extrapolating the limits of this research, it can be hypothesized that the barriers to vegetarian meals are more conceptual, behavioral, cultural, and systemic than sensorial [11,12]. When the simple idea of a pea burger is less liked than of a beef burger [31] and meat attachment has been shown to influence how much consumers like plant-based alternatives [16], it is fair to question if an inherently sensory inferiority is a major limiting factor for plant-based alternatives [9]. Measuring consumer attitudes towards vegetarian food was not part of the goals of these two studies, but “vegetarian” being the most marked term on CATA shows how important the nature of the meal was to participants. Even when most participants were Swedish young adults, who are generally more open to plant-based protein [9], and the dishes were overall liked (all means were above five on a scale from “1—Very bad” to “7—Very good”). Naturally, the discounted price and familiarity, indulgence, and comforting aspects of a burger must have played a role in the overall liking too [32].

This research is limited to its samples (gray pea burgers), variables (grilling, adding MSG and spices), context (a subsidized “burger week” at a campus restaurant), conditions (participants were free to eat anywhere, anyhow, with any drink) and participants (mostly young students at a Swedish university). Future research should test the variables with different dishes, contexts, and participants to assess the generality of the findings. It should also further explore the importance of texture in liking of vegetarian substitutes, other umami enhancers, and include physical–chemical analyses. Expanding the discussion, more research is needed to assess how consumers’ attitudes and preconcepts limit their liking and preference for vegetarian meals. Future studies should challenge the popular assumption that there is an innate sensorial gap between omnivore dishes and vegetarian dishes, such as lack of umami, Maillard or complexity. Cultural barriers, including lack of motivation from cooks and skepticism from consumers, may play a bigger role in the rejection of vegetarian meals [33,34].

## 5. Conclusions

Two studies were conducted to test umami, Maillard reaction, and flavor complexity as flavor enhancers of a gray pea burger in a real consumption setting. The results show that participants perceived sensory differences among the samples, but overall liking and WTB were not consistently or strongly influenced by them. Even though consumer research with preference and attitude questionnaires indicates that sensory attributes play a major role in their food choices, there can be many other unconscious important drivers such as prejudice against vegetarian meals. Results suggest that future research could probably focus more on serving tasty meals and overcoming cultural barriers than perfectly mimicking meat.

## Figures and Tables

**Table 1:** Demographic profile of the participants in Studies 1 and 2.

	Control 1 (*n* = 103)	Maillard (*n* = 95)	Umami (*n* = 96)	Complex 1 (*n* = 101)	Total Study 1 (*n* = 395)	Control 2 (*n* = 112)	Complex 2 (*n* = 105)	Total Study 2 (*n* = 217)
Student	87 (84%)	84 (88%)	80 (83%)	92 (91%)	343 (87%)	94 (84%)	94 (90%)	188 (87%)
Non-student	16 (16%)	11 (12%)	16 (17%)	9 (9%)	52 (13%)	18 (16%)	11 (10%)	29 (13%)
Female	61 (59%)	59 (62%)	49 (51%)	56 (55%)	225 (57%)	55 (49%)	50 (48%)	105 (48%)
Male	42 (41%)	36 (38%)	47 (49%)	45 (45%)	170 (43%)	56 (49%)	54 (51%)	110 (51%)
Non-binary	0	0	0	0	0 (0%)	1 (1%)	1 (1%)	2 (1%)
16–25 y.o.	73 (71%)	70 (74%)	69 (72%)	80 (79%)	292 (74%)	80 (71%)	80 (76%)	160 (74%)
26–35 y.o.	14 (13%)	17 (18%)	15 (16%)	13 (13%)	59 (15%)	21 (19%)	16 (15%)	37 (17%)
36–45 y.o.	10 (10%)	2 (2%)	6 (6%)	2 (2%)	20 (5%)	5 (4%)	4 (4%)	9 (4%)
46–55 y.o.	6 (6%)	2 (2%)	2 (2%)	4 (4%)	14 (3%)	3 (3%)	3 (3%)	6 (3%)
≥56 y.o.	0	4 (4%)	4 (4%)	2 (2%)	10 (3%)	3 (3%)	2 (2%)	5 (2%)

**Table 2:** Composition of the patties from Studies 1 and 2 in percentage of the total weight before cooking.

	Sample	Grilled	Gray Pea	Carrot	Onion	Oat	Potato Flour	Garlic	Salt	MSG	Spices	Liquid Smoke
Study 1	Control	No	77%	7%	7%	4%	2%	1%	1%	–	–	–
	Maillard	Yes	77%	7%	7%	4%	2%	1%	1%	–	–	–
	Umami	No	77%	7%	7%	4%	2%	1%	0.5%	0.5%	–	–
	Complex	Yes	77%	7%	7%	4%	2%	1%	0.5%	0.5%	–	–
Study 2	Control 2	No	91%	–	–	5%	3%	–	1%	–	–	–
	Complex 2	Yes	74%	7%	7%	4%	2%	1%	0.5%	0.5%	1%	1%

**Table 3:** Average liking and standard deviation of the potatoes and the burger on a 7-point Likert scale, the willingness to buy (WTB) on a 5-point scale, and frequency of selection of CATA terms for each of the four samples in Study 1. Values marked with the same subscribed letter in a line are not significantly different. * *p* < 0.05, ** *p* < 0.01.

	Control (*n* = 103)	Maillard (*n* = 95)	Umami (*n* = 96)	Complex (*n* = 101)
Liking potato	5.93 ± 1.03	5.65 ± 1.16	5.85 ± 1.26	5.68 ± 1.07
Liking burger *	5.09 ± 1.21 ^b^	5.58 ± 1.19 ^a^	5.25 ± 1.29 ^ab^	5.08 ± 1.44 ^b^
WTB	3.06 ± 1.26	3.43 ± 1.09	3.17 ± 1.41	2.99 ± 1.36
*CATA frequency*				
Simple	20	22	19	22
Complex	4	5	4	10
Meaty	4	1	5	5
Filling	12	14	13	15
Boring	15	16	18	15
Fun	28	19	21	27
Dry **	28 ^a^	15 ^b^	27 ^ab^	12 ^c^
Juicy	12	12	16	20
Soft	25	28	23	24
Crispy	14	16	9	10
Umami	6	8	4	4
Salty	5	4	3	2
Roasted	9	7	6	4
Vegetarian *	61 ^ab^	44 ^b^	52 ^ab^	65 ^a^
Healthy	32	33	34	43
Luxurious	5	2	3	4
Cheap	29	25	24	27
Spiced	12	12	9	11
Smoky	15	15	11	14
Nutty	18	11	12	11
Long aftertaste *	13 ^a^	6 ^ab^	4 ^b^	6 ^ab^
Sustainable	30	25	28	26

**Table 4:** Average liking and standard deviation of the potatoes and the burger on a 7-point Likert scale, the willingness to buy on a 5-point scale, and frequency of selection of CATA terms for each of the two samples in Study 2. * *p* < 0.05, ** *p* < 0.01, *** *p* < 0.001.

	Control 2 (*n* = 112)	Complex 2 (*n* = 105)
Liking potato	5.73 ± 1.14	5.71 ± 1.25
Liking burger	5.38 ± 1.25	5.33 ± 1.43
WTB	3.53 ± 1.33	3.35 ± 1.26
*CATA frequency*		
Simple	27	18
Complex	6	3
Meaty	3	2
Filling	13	20
Boring	12	10
Fun	24	30
Dry	21	14
Juicy	22	23
Soft	21	29
Crispy *	10	19
Umami	6	4
Salty	6	2
Roasted	8	12
Vegetarian	52	52
Healthy	37	34
Luxurious	2	4
Cheap	27	26
Spiced **	17	32
Smoky	12	18
Nutty	24	14
Long aftertaste ***	3	19
Sustainable	28	20

## Data Availability

Data collected for this study will be publicly available under CC BY 4.0 license at https://doi.org/10.60689/eg10-xj20.
